# DVID: Distributed Versioned Image-Oriented Dataservice

**DOI:** 10.3389/fncir.2019.00005

**Published:** 2019-02-05

**Authors:** William T. Katz, Stephen M. Plaza

**Affiliations:** Janelia Research Campus, Howard Hughes Medical Institute, Ashburn, VA, United States

**Keywords:** versioning, connectomics, EM reconstruction, dataservice, big data, datastore, collaboration, distributed version control

## Abstract

Open-source software development has skyrocketed in part due to community tools like github.com, which allows publication of code as well as the ability to create branches and push accepted modifications back to the original repository. As the number and size of EM-based datasets increases, the connectomics community faces similar issues when we publish snapshot data corresponding to a publication. Ideally, there would be a mechanism where remote collaborators could modify branches of the data and then flexibly reintegrate results via moderated acceptance of changes. The DVID system provides a web-based connectomics API and the first steps toward such a distributed versioning approach to EM-based connectomics datasets. Through its use as the central data resource for Janelia's FlyEM team, we have integrated the concepts of distributed versioning into reconstruction workflows, allowing support for proofreader training and segmentation experiments through branched, versioned data. DVID also supports persistence to a variety of storage systems from high-speed local SSDs to cloud-based object stores, which allows its deployment on laptops as well as large servers. The tailoring of the backend storage to each type of connectomics data leads to efficient storage and fast queries. DVID is freely available as open-source software with an increasing number of supported storage options.

## 1. Introduction

Generation of a connectome from high-resolution imagery is a complex process currently rate-limited by the quality of automated segmentation and time-consuming manual “proofreading,” which entails examination of labeled image volumes and correction of errors (Zhao et al., 2018). Advances in the acquisition and segmentation of high-throughput volume electron microscopy (VEM) create larger data sets (Kornfeld and Denk, 2018) that stress data management tools due to the volume of data, the need to support proofreading as well as automated, high-throughput batch operations, and the sharing and integration of results from different research groups. While many data distribution systems focus on large numbers of relatively small datasets or file-based distribution (Dutka et al., 2015; Viljoen et al., 2016), VEM reconstructions are not easily distributed and usable to researchers through file distribution. For teravoxel to petavoxel datasets, centralized data services can provide low latency access to areas of interest without requiring the download of much larger volumes of data (Saalfeld et al., 2009; Burns et al., 2013; Haehn et al., 2017; Kleissas et al., 2017).

As reconstructions increase in both number and size, more data will be published after automated segmentation and a decreasing portion of the reconstructions will be manually proofread due to the flood of new data. Research groups around the world should be able to download regions of interest and edit them locally to further improve reconstructions to higher levels of accuracy. However, no connectomics data system exists that allow remote or post-publication editing on data copies with the option to easily integrate these changes with other copies, including the original, centralized data repository. Distributed version control systems for software, like git and the collaborative website github.com (Blischak et al., 2016), provide workflow examples of how scientific data could be shared, forked, and collaboratively edited, even if git is not a viable system for handling large VEM reconstructions.

Connectomics data is also quite heterogeneous. In addition to the large volumes of grayscale and segmentation images, there can be agglomeration information in the form of supervoxels and merge/split trees as well as synapse and workflow data useful to managing the reconstruction process. Low latency retrieval of neuron data will probably require denormalizations of data such that segmentation is not only held across multiple resolutions but also separated into neuron-specific sparse volumes (i.e., compressed binary representations that can span large volumes). The various forms of data can be mapped onto different storage systems based on requirements for data size, latency, and cost per terabyte. Data services should be available in isolated, small compute environments like laptops as well as institutional clusters and multi-region clouds.

Over time, connectomics researchers will create a variety of tools that need access to the data despite possible changes in how the data is stored. A high-level Science API, focused on connectomics operations, can shield clients from infrastructure changes and allow easier support of multiple tools.

**Figure 1:**
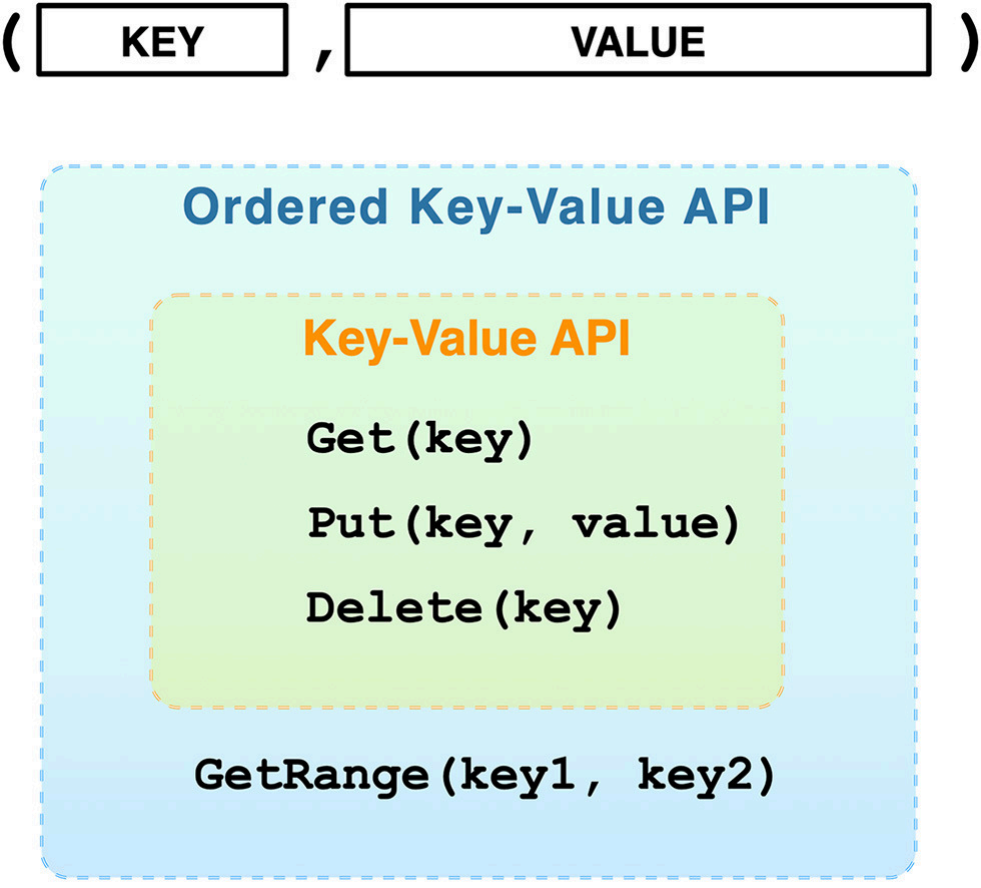
Key-value stores are among the simplest databases with few operations. Because of their simplicity, many storage systems can be mapped to key-value interfaces, including file systems where the file path is the key and the value is the file data.

DVID^1^ has made several contributions to the state of the art. First, it provides a simple mechanism that efficiently adds branched versioning to storage systems that provide key-value store interfaces (Figure 1). Our branched versioning system permits instantaneous viewing of older versions, novel workflows for training proofreaders, and git-like methods of distributing data and updating remote stores. It allows one to treat committed nodes as immutable data and leaf nodes as mutable. Under this model, most of the connectome data will be immutable. The use of storage via a key-value interface allows us to exploit a spectrum of caching and storage systems including in-memory stores, embedded databases, distributed databases, and cloud data services. By partitioning data into key-value pairs, we efficiently handle versioning by only storing new key-value pairs covering modifications and not copying all data for each version.

DVID introduces the idea of typed data instances that provide a high-level Science API, translate data requirements to key-value representations, and allow mapping types of data to different storage and caching systems. The Science API provides a reliable connectomics interface for clients and frees them from requiring specific database technology or reimplementing domain-specific processing. The mapping system allows DVID to assign some data to very low-latency storage devices like Non-Volatile Memory Express (NVMe) SSDs while exploiting cheap, petabyte-scale cloud stores and efficient caching systems for immutable grayscale data.

DVID provides a publish/subscribe mechanism for messaging between data types so changes in one data instance can trigger modifications in another. For example, if a segmentation changes, associated synapses will be automatically modified so that requests for all synapses in a particular label will be correct.

DVID was introduced in 2013 as an open-source project and became the principal data system for the FlyEM team at Janelia Research Campus for several of the largest, dense VEM reconstructions done to date. Over the course of its use, we added a number of features driven by reconstruction demands including multi-scale segmentation, regions of interest, automatic ranking of labels by synapse count, supervoxel and label map support that provides quick merge/split operations, and a variety of neuron representations with mechanisms for updating those denormalizations when associated volumes change. This paper discusses some of the issues and interesting benefits that we discovered in using a branched versioning system for our research.

## 2. System Design

The DVID system is a highly customizable, open-source dataservice that directly addresses the issues encountered by image-driven connectomics research. DVID provides versioning and distribution inspired by software version control systems, customizable domain-specific data types (e.g., grayscale and label volumes, synapse annotations) accessible via a HTTP API, and flexibility in choosing underlying storage engines, allowing its use on laptops, institutional clusters, and the cloud.

DVID persists data through an abstract key-value interface that is satisfied by a number of swappable storage engines. We started with a key-value interface because (1) there are a large number of high-performance, open-source caching and storage systems that match or can be reduced to a key-value API, (2) the surface area of the API is very small, even after adding important cases like bulk loads or sequential key read/write, and (3) versioning can be easily added by modifying keys to incorporate a version identifier.

**Figure 2:**
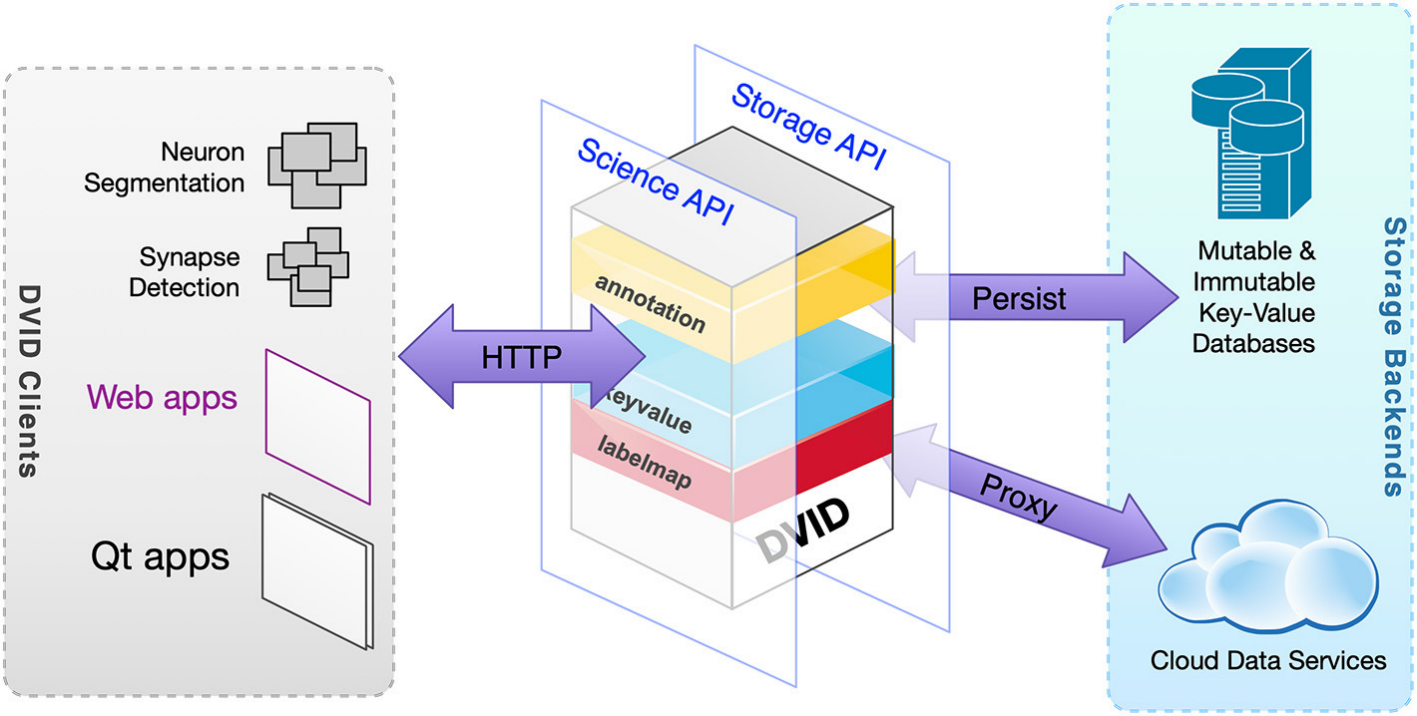
High-level view of DVID. Data types within DVID provide a Science API to clients while transforming data to meet a primarily key-value Storage API or proxy data to a connectomics service.

From a user's perspective, the DVID system can be described through its two major interfaces. The first is a client-facing Science API that provides a rich set of connectomics operations through a REST (Level 2 of Richardson Maturity Model^2^) HTTP API (Table 1). The second is a Storage API that provides a limited set of key-value operations (Figure 2).

**Table 1:** Sample of science HTTP API.

**Datatype**	**Endpoint (URL starts with */api/node/f8a0…*)**	**HTTP action**
**Labelmap**	/*name*/raw/128_128_128/0_0_0	**GET** returns and **POST** stores
		128^3^ voxel subvolume at offset (0, 0, 0).
	*/name*/specificblocks?blocks=23,23,10,23,24,10	**GET** returns compressed block data
		for blocks (23, 23, 10) and (23, 24, 10).
	/*name*/label/100_100_47	**GET** returns JSON for the *uint64* label
		at voxel (100, 100, 47).
	/*name*/size/3171	**GET** returns JSON for the number of
		voxels in label 3171.
	/*name*/sparsevol/3171?format=rles&minz=60	**GET** returns run-length encoded list
		of voxels with *z*≥60 in label 3171.
	/*name*/merge	**POST** merges labels given in POSTed
		JSON array *[target,label1,label2,…]*.
	/*name*/split/3171	**POST** splits label 3171 using a POSTed
		sparse volume.
**Annotation**	/*name*/elements	**POST** stores 3D point annotations given in
		POSTed JSON.
	/*name*/elements/200_200_200/0_0_0	**GET** returns JSON of annotations within
		200^3^ voxel subvolume at offset (0, 0, 0).
	/*name*/move/38_21_33/46_23_35	**POST** moves the annotation at voxel
		(38, 21, 33) to (46, 23, 35).
	/*name*/label/3171	**GET** returns JSON of annotations in voxels
		with label 3171.
**Keyvalue**	/*name*/key/somedata	**GET** returns and **POST** stores arbitrary
		data with key “*somedata*.”
	/*name*/keyvalues	**GET** returns and **POST** stores arbitrary
		key-value data using protobuf serialization.
	/*name*/keyvalues?jsontar=true	**GET** returns a tarball of key-value data for
		keys given in the query body as a JSON
		string array.

*Each datatype implements its own HTTP endpoints although similar datatypes (e.g., ones dealing with image volumes) can reuse interfaces like the first “raw” endpoint*.

From a developer's perspective, pluggable data type code packages (e.g., a **uint8blk** type that supports *uint8* image volumes like VEM grayscale data) expose a data type-specific HTTP/RPC API on the user-facing side and processes data in the form of key-value pairs that get persisted through the storage interface. The storage interface is handled by pluggable storage engine code packages that can obtain version and data instance IDs from the key and store the key-value pair in a reasonable way for the particular storage system. Data types also can be constructed that simply proxy requests to a backend service like *bossDB* (Kleissas et al., 2017) at the cost of versioning.

### 2.1. Example Usage

Before detailing how DVID implements the Science API and versioned data, we will describe how DVID is used in an example reconstruction and connectome analysis workflow.

DVID administration can be performed through a DVID command in a terminal, sending a HTTP request through tools like curl or httpie^3^, or using the *DVID Console* web application^4^. Using one of those three methods, we first create a new repository, and then add a **uint8blk** data instance called *grayscale* and a **labelmap** data instance called *segmentation*.

Due to the large scale of our image volumes, FlyEM employs python scripts that load strips of grayscale data using HTTP POST requests to the *grayscale* instance. These HTTP requests are typically handled by *libdvid*, a C++ library with python bindings^5^. Similarly, scripts load the automatically segmented label data (64-bit unsigned integer per voxel) (Januszewski et al., 2018) into the *segmentation* instance using strips of highly-compressed DVID blocks (see section 2.5). Both grayscale and segmentation voxel data can be loaded into DVID, letting DVID do the image pyramid generation as well as the label indexing (i.e., determining the blocks spanned by each label). However, for very large volumes, it is more efficient to offload the image downsampling and label indexing to a cluster and then directly ingest the results. We have published Spark (Zaharia et al., 2010) tools that can be used for large-scale processing^6^.

After ingestion of the image volume and segmentation, we commit (lock) the root version and create a new version for our manual proofreading. Additional data instances are typically created, such as a *synapses* instance of **annotation** to hold synapse point annotations and various **keyvalue** instances to hold proofreader assignments, generated skeletons, and other data useful to the various clients and scripts used for reconstruction and connectome analysis. In each case, python, C++, or Javascript clients connect with DVID through the languages' built-in HTTP library or intermediate libraries like *libdvid*.

Proofreaders use tools like NeuTu (Zhao et al., 2018), Neuroglancer^7^, and various scripts to edit the segmentation, view 2D image sections, 3D sparse volumes, meshes, and skeletons, and manage data necessary for our proofreader workflows. HTTP traffic to DVID can easily exceed 100,000 requests per minute and include server metadata queries that return within microseconds as well as sparse volume requests for massive bodies that take tens of seconds.

At some point in time, we may decide to create a snapshot of all the data so we commit the current version and create a new one. The state of the data at the time of that commit will always be available for instantaneous viewing.

### 2.2. Versioned Data

Versioning can be modeled in at least two ways: branched versioning using a directed acyclic graph (DAG) as in the git software version control system, or a linear timeline that can be thought of as one path through the DAG. Current connectomics data services use no versioning or linear versioning where there is one head, the current state. The underlying storage can be optimized for access of the current state while any changes are recorded into a log, which will be likely accessed less frequently than the head (Al-Awami et al., 2015).

A DAG-based approach is more powerful, allowing branching and merging, and has been shown to be very effective for collaborative efforts like distributed software development. While branched versioning is already showing utility for proofreader training as described below, we believe its utility will be more obvious as published reconstructions increase in both number and size and the portion of manually proofread data decreases. As discussed in Future Work, specialists in various neural circuits will be able to improve reconstruction accuracy of published regions, maintain their own private branch until publishing results, and then optionally merge edits back into the central repository.

The DAG, in one way or another, will be dictated by post-publication manual proofreading as well as any collaborative editing involving decentralized data storage. Each edited clone is essentially a branch, even if described as a control layer over linear versioning, and attempts to merge results require a DAG for provenance tracking. Aside from edits due to continued reconstruction improvements, full-fledged branching and merging is also important for collaborative data analysis (Huang et al., 2017). The drawback of a DAG is its complexity, but even if a versioning system uses a DAG internally, its clients could show a single selected branch unless handling operations that need to expose that complexity.

Distributed software version control systems like git use the nodes in a DAG to represent each committed version of data. A commit is a snapshot of data at that time, and as such, would include an accumulation of changes since the last commit. Provenance is kept at the commit level, not the change level, so if a line in a file were changed three times since the last commit, only its final state would be recorded and not the individual changes between the commits. While it would be nice to have a complete record of all changes to data, there could be a significant storage overhead for storing every change regardless of its importance. For this reason, fine-grain provenance, if desired, is delegated to the data type implementation while commit-level versioning is standard for all versioned data instances. Many data types publish each mutation to an Apache Kafka system, a distributed logging system that can be used to inform other services of changes to data (Wang et al., 2015). In addition to Kafka logging, the data type **labelmap** always provides fine-grained provenance by logging all mutation operations like merges and splits to an append-only file. This log is used on server restart to populate an in-memory label map, which provides supervoxel id to agglomerated label mappings.

At its core, DVID adopts the DAG view of versioning used by software version control systems like git. Unlike git, DVID partitions data not in files but in data instances of a data type, for example, there could be *segmentation-param1* and *segmentation-param2* instances of data type **labelmap**, which supports label volumes and label-specific sparse volume retrieval and operations. DVID also allows access to any version of the data at any time.

A dataset in DVID is described as a “repository” and corresponds to a single DAG. Each node is a version identified by a RFC4122 version 4 UUID^8^, a 32 character hexadecimal string that can be generated locally and is unique globally. Datasets are typically identified by the UUID of the root. At each node of the DAG, users can store a description similar to git's commit message as well as append to a node-specific log.

DVID requires each branch of the DAG to have a unique string name. By default, the root node is part of the “master” branch that uses the empty string for a name. For each committed node, we can create one child that extends the parent branch or any number of children with new branch names.

**Figure 3:**
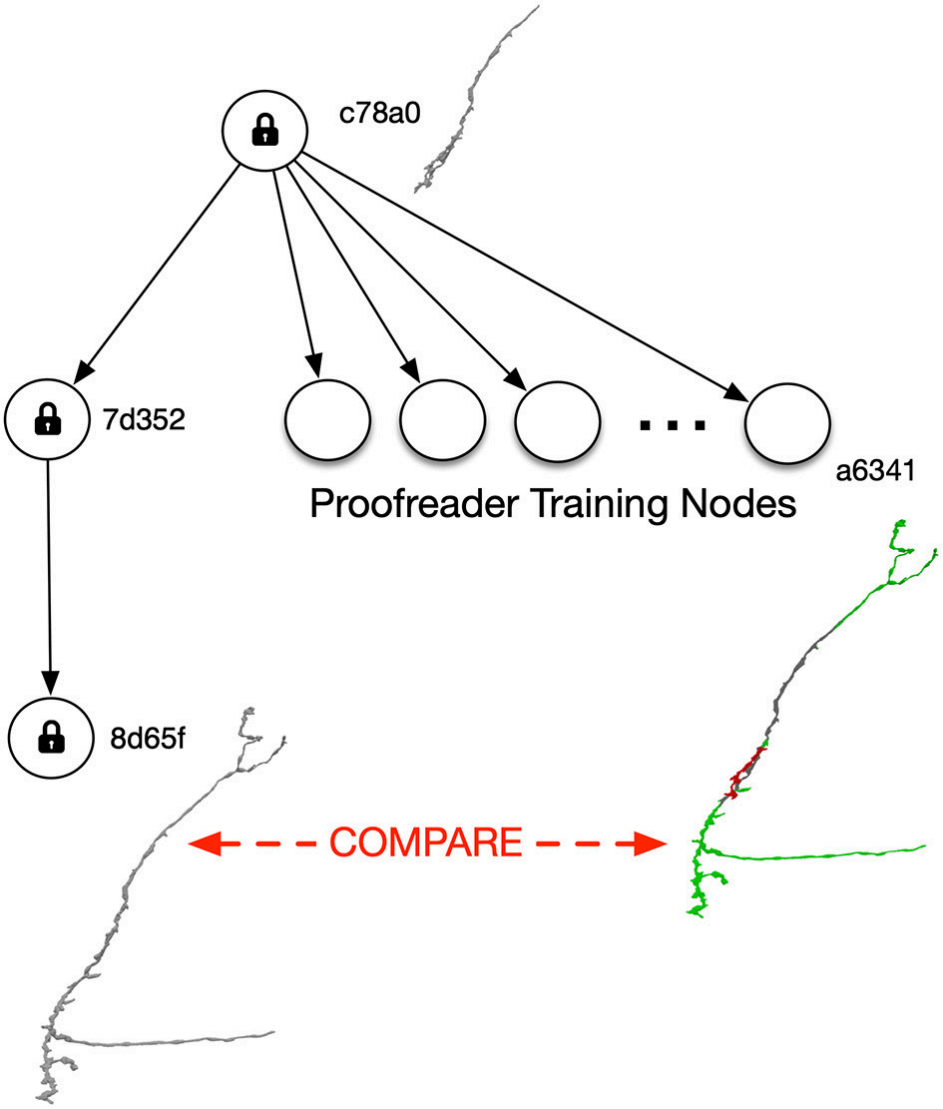
Versioning can help train proofreaders without requiring any changes to proofreading tools. After full proofreading (version 8d65f), an interesting neuron is selected and its precursor at the root version c78a0 is assigned for training. Each trainee gets her own branch off the root version, and the reconstructed neuron (e.g., the one depicted in training version a6341) can be compared to version 8d65f.

The ability to easily branch and handle distributed editing is a significant advantage of a DAG approach. Proofreaders can branch their own versions to allow training and testing (Figure 3). As described below, branching requires little additional storage cost since only modifications need to be stored. Also, no modification to tools are required since DVID clients can simply specify the UUID of a training version and leave the current “master” data unaffected.

**Figure 4:**
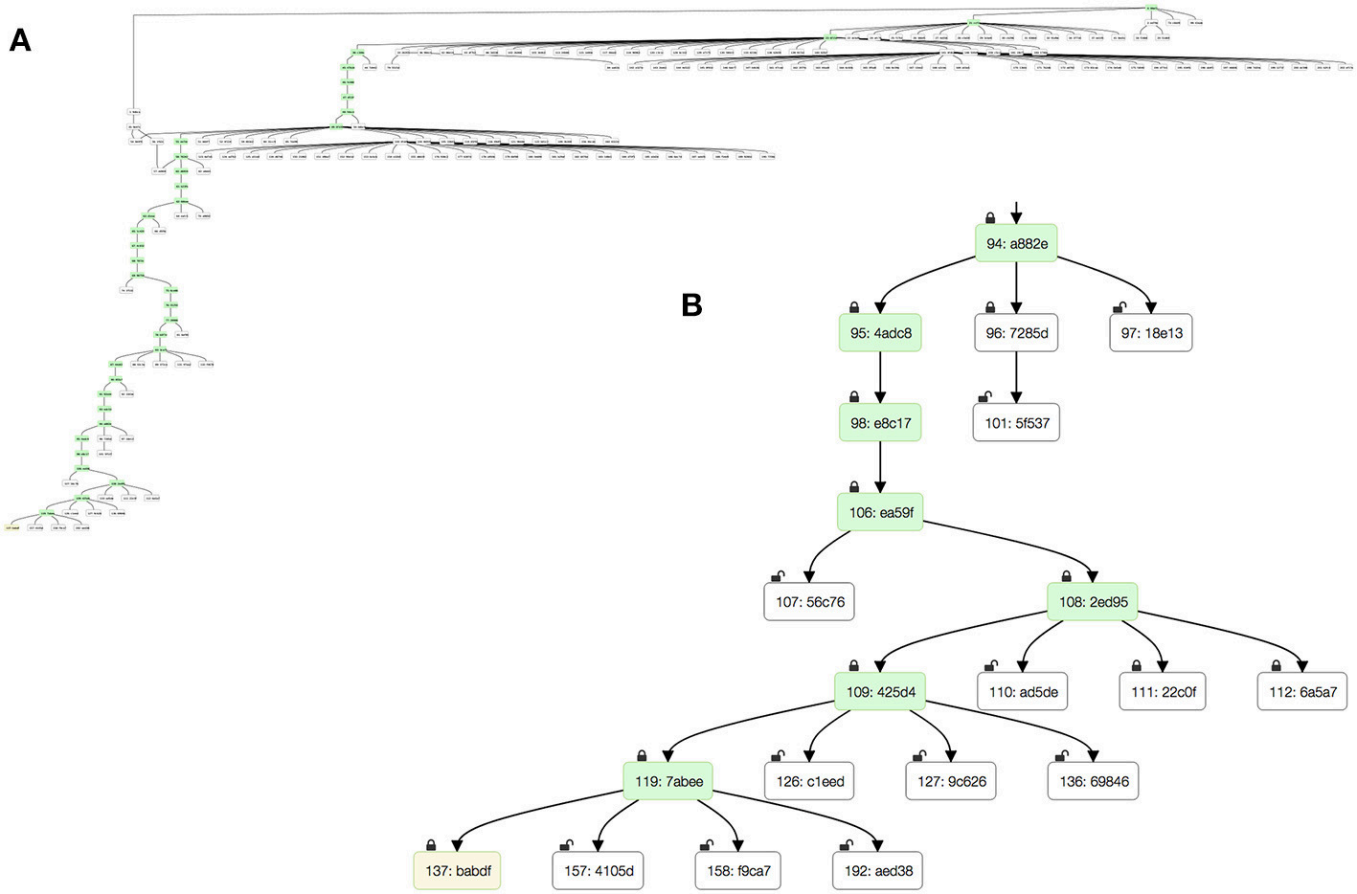
The version DAG of the mushroom body reconstruction as seen through the *DVID Console*'s DAG viewer. Snapshots show **(A)** zoomed out view showing extent of DAG with significant proofreader training branches near root, and **(B)** blown up view of leaf at bottom left. Green nodes highlight the “master” branch while the yellow leaf node is the current production version.

Over the past 3 years, the FlyEM team has used DVID during reconstructions of seven columns of medulla (Takemura et al., 2015) and the mushroom body (Takemura et al., 2017) of Drosophila. The reconstruction process produced large DAGs with regions of heavy branching due to proofreader training or experimental edits (Figure 4). DVID provides an extensive HTTP API for clients to download server state and dataset metadata, including the DAG. The *DVID Console* web application provides a simple view of the master branch, and an alternative version allows viewing of the full DAG as well as the ability to click any node to view the log and data instances associated with that node.

The DAG is useful for quality control and being able to look back to previous states of our dataset as well as the comments attached to it. If mistakes were made, we can determine where they were introduced. While viewing historical data only requires versioning, not necessarily branched versioning, its ease of use requires a data service that can display all versions without a significant time delay.

### 2.3. Branched Versioning of Key-Value Data

DVID implements branched versioning over different types of key-value data by (1) keeping metadata, including the version DAG, in memory and (2) extending keys to include data and version identifiers. When data is modified in an uncommitted, open node, the data type implementation retrieves and stores key-value pairs as needed while the core DVID system modifies the keys to include identifiers for the particular data instance and the version. We want to emphasize that modifications in a version usually only affect a subset of key-value pairs. For example, the **labelmap** data type partitions 3D space into small blocks (typically 64 × 64 × 64 voxels) such that each block is a single key-value pair. Modifications of the label volume in a new version requires only storing the affected blocks, and key-value pairs corresponding to untouched areas will be inherited by ancestors as described below.

**Figure 5:**
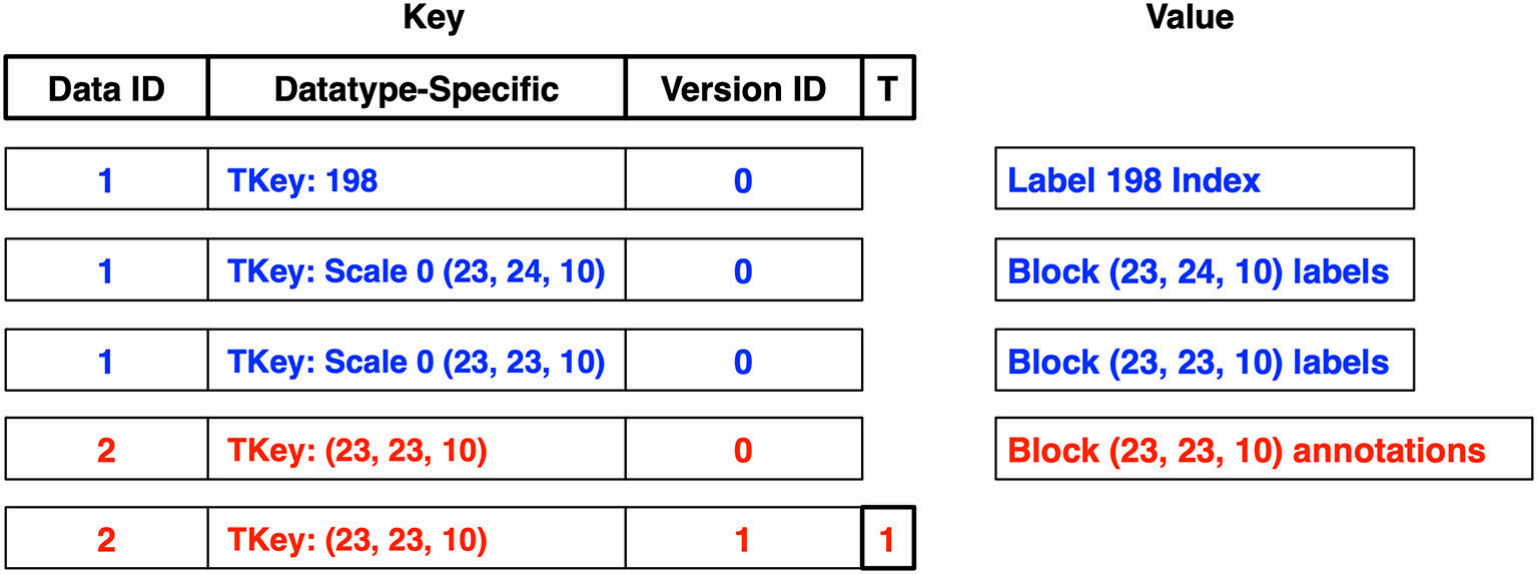
Each data type persists data using datatype-specific key-value pairs. Key-value pairs for two data instances are shown: a **labelmap** instance (data id 1) in blue and an **annotation** instance (data id 2) in red. The datatype-specific component of a key (**TKey**) could be a block coordinate for a block of voxels. DVID then wraps this **TKey**, prepending a short data instance identifier and appending a version identifier. A tombstone flag (**T**) can mark a key-value as deleted in a version without actually deleting earlier versions, as shown for the last key, which marks the deletion of annotations in block coordinate (23, 23, 10) in version 1.

The core DVID storage package uses a key composed of a data instance identifier, the datatype-specific key, a version identifier, and a tombstone marker, in that order by default as shown in Figure 5. A data instance can insert multiple classes of key-value pairs, each with different formats of datatype-specific keys and corresponding values. For example, the **labelmap** and **labelarray** data types (described in more detail in section 2.5) use two classes of key-value pairs: (1) blocks or cuboids of label data where the datatype-specific key has a scale integer prepended to the ZYX block coordinate, and (2) label indices where the datatype-specific key is simply a 64-bit unsigned integer label and the value describes the blocks containing the label in question.

DVID maintains a mapping of globally unique 128-bit data instance and version UUIDs to unsigned 32-bit integers solely to decrease key sizes. The 32-bit identifiers are *server-specific* since these identifiers could collide with identifiers in remote DVID servers as new data instances and versions are added locally and remotely. When key-value pairs are exchanged with remote DVID servers, the source server identifiers are converted to remote server identifiers by comparing the globally unique data instance and version UUIDs.

In Figure 5, two data instances are shown: a **labelmap** instance (data id 1) and an **annotation** instance (data id 2). The **labelmap** instance has key-value pairs for label 198's index and two label blocks in version 0, and the **annotation** instance holds a single block of annotations. A tombstone flag can mark a key-value as deleted in a version without actually deleting earlier versions, as shown for the last key, which marks the deletion of annotations in block coordinate (23, 23, 10) in version 1. The annotations for that block still exist in version 0 since a non-tombstoned key exists.

#### 2.3.1. Overhead of Versioning

**Figure 6:**
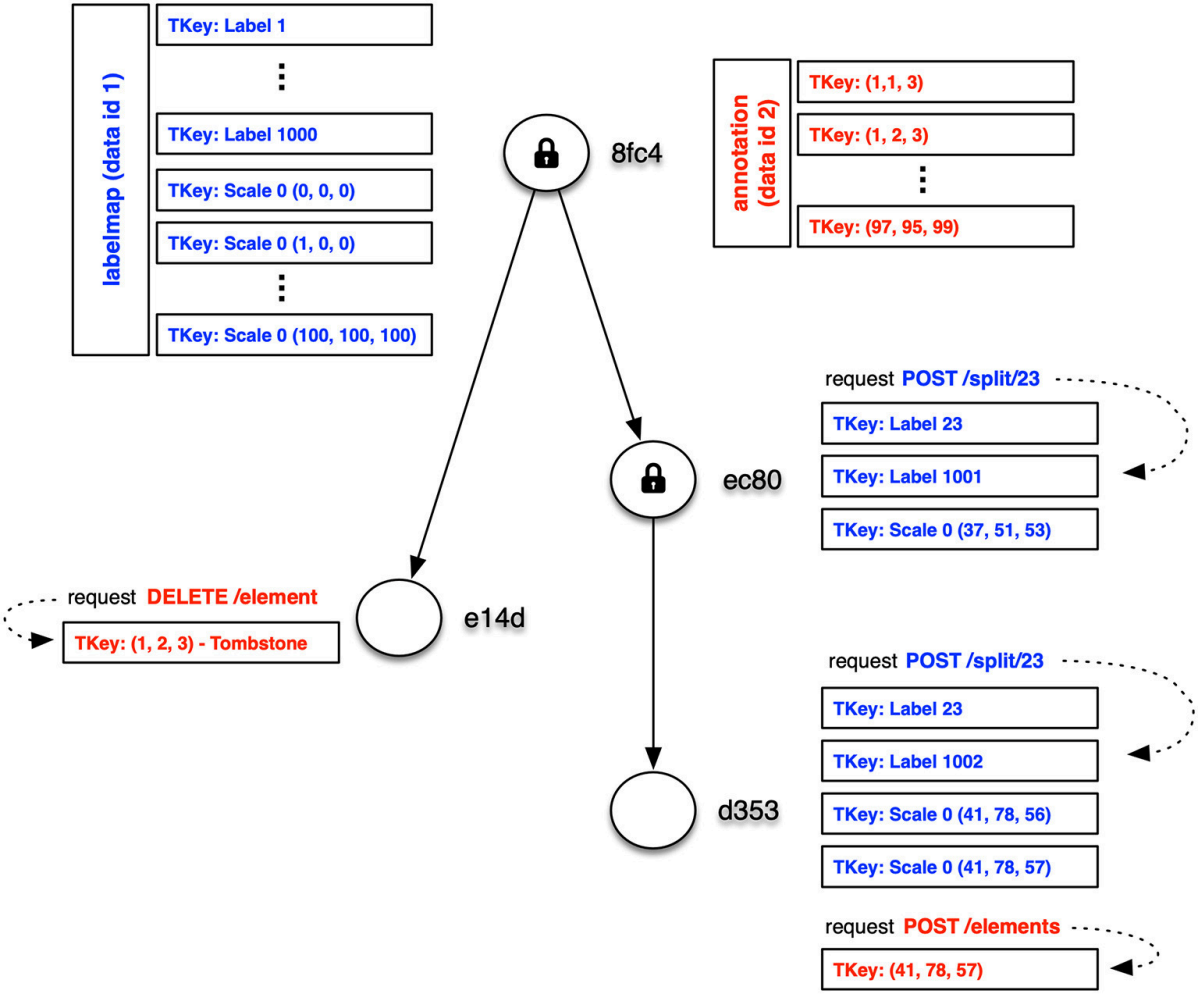
Simple example of distribution of key-value pairs across the nodes of a DAG (only keys shown). In this example, segmentation and synapse data for a 6,400^3^ voxel volume with 1,000 labels is stored in **labelmap** (blue) and **annotation** (red) instances at the root version *8fc4*. The majority of key-value pairs are ingested at the root and only modified key-value pairs need to be stored for later versions. Several mutation requests are shown with their modified key-value pairs.

Figure 6 shows how key-value pairs from different data instances can be distributed across a DAG. In this example, segmentation data for a 6,400^3^ voxel volume with 1,000 labels is stored in a **labelmap** instance (shown in blue) at the root version *8fc4*. The segmentation requires one million key-value pairs for label block data and another 1,000 key-value pairs for the label indices. Additionally, synapse 3D point data is stored in an **annotation** instance (shown in red). The annotation data requires key-values for only the blocks containing synapses.

The majority of key-value pairs are ingested at the root of the DAG and only modified key-value pairs need to be stored for later versions. In Figure 6, three additional versions have been created. In version *e14d* the synapse annotations for block (1, 2, 3) was deleted by storing a tombstone key. Clients that access version *e14d* can access all the data stored in the root version with the exception of synapses in that one block. In version *ec80* we splitted a small fragment from label 23, which required modification of the label block (37, 51, 53) containing that fragment as well as the key-value pairs for the label 23 index and a new label 1001 index for the split voxels. In version *d353* we splitted from label 23 another small fragment that spanned two blocks, and we added a new synaptic annotation to that region. These operations required the addition of a few more key-value pairs that take precedence over earlier key-value pairs.

Teravoxel datasets can require more than a hundred million key-value pairs, depending on the chosen size of a labelmap instance's block, and mutations to individual neurons will alter a very small percentage of the key-value pairs. So rather than duplicating unmodified data for each commit or snapshot, the decomposition of data into more granular key-value pairs allows efficient versioning.

One issue is determing the relevant key-value pairs for any version of a data instance. As we saw in Figure 5, the version identifier is typically appended to the type-specific key, which leads to different versions of a type-specific key to be grouped together in systems that order keys. Key-ordering occurs in many popular key-value databases, particularly those that employ log-structured merge trees (O'Neil et al., 1996) like leveldb^9^, RocksDB^10^, and newer systems based on WiscKey (Lu et al., 2016). These databases provide range queries (see Ordered Key-Value API in Figure 1). Because the versions of a key-value pair are grouped together, we can use range queries to *sequentially* load the keys or key-value pairs into memory, and then use our in-memory DAG to select the most recently stored ancestor of our desired version. Sequential access provides significant speed advantages across rotational disks, solid-state drives, and even in-memory storage (Jacobs, 2009).

Returning to the example of 3d label data types like **labelmap**, if a block was modified in *N* versions of that data instance, it will require *N* key-value pairs. Support of versioning for that particular block will incur the overhead of now reading *N* key-value pairs instead of just one, as well as the time to calculate the closest stored version using our in-memory DAG. However, the cost of handling unnecessary versions is countered by the significant speeds of both disk-based and in-memory sequential access. The number of modified versions for a block should be small because most regions are not constantly modified due to manual proofreading. As seen in section 2.5, the **labelmap** data type uses relatively static voxel labels (i.e., supervoxel identifiers) and maintains supervoxel-to-neuron mappings as neurons are split and fragments are merged.

#### 2.3.2. Support for Non-ordered Key-Value Stores

We have also built support for non-ordered key-value stores where range queries are either costly or not available. Google Cloud Storage^11^ can be viewed as a distributed, petabyte-scale key-value store that supports conditional writes as well as key prefix searches, which could be used to implement range queries. Unfortunately, these key prefix searches introduce significant latency for each data request. We observed that key spaces can be divided into two categories: a *computable* key space where valid keys can be computed (e.g., the **uint8blk** data type stores blocks of grayscale with block coordinate keys, easily calculated for requested 3D subvolumes) or a *non-computable* key space where arbitrary keys are used (e.g., the **keyvalue** data type that allows user-specified keys).

In practice, we only use Google Cloud Storage for data types with computable key spaces. Even with this restriction, versioning requires range queries or speculative queries on all possible key versions when retrieving a particular key-value pair (*kv*).

To solve this problem, we introduced a novel strategy to eliminate costly key searches or the need to separately maintain an index of stored keys. For each type-specific key, we maintain a single, versionless *kv* that stores the keys for all versions and the highest priority *kv*, which comes from the most recent *kv* in the *master* branch or, if no version of this key exists in *master*, the most recent *kv* of any branch. Writes of a versioned *kv* start with a conditional write to the versionless *kv*. If the conditional write succeeds, it is the first write of any version to this key and we are done. If the conditional write fails, we read the versionless *kv* and compare the new *kv* to its stored *kv*. If the new key has higher priority, the new value evicts the stored *kv* to its own versioned *kv*. If the new key has lower priority, we write the new versioned *kv* and append its version to the list of all versions stored in the versionless *kv*. With this approach, we achieve the following properties:

- Writes of the first version of any type-specific key are as fast as an unversioned one. Since data destined for this type of store tends to be immutable, write performance is not degraded in most cases.
- Any read of the highest priority *kv* will be as fast as an unversioned read.
- Any read of a lower priority *kv* will require reading the list of versions in the versionless *kv*, finding the version closest to the desired version using the version DAG, and then reading that versioned *kv*.

As shown by the Google Cloud Storage example above, a DVID storage engine can tailor the implementation of versioned storage to the capabilities of a storage system. DVID storage engines can also override the default key and form a version-first representation (Bhardwaj et al., 2014) if it is more advantageous to group all *kv* by version instead of by type-specific key. This approach can be particularly useful for optimizing transmission of *kv* corresponding to a subset of versions, as would happen when synchronizing with a remote DVID server. A proposed DVID store, described in Future Work, takes this approach since we can create a compact, in-memory index of all stored keys in committed, immutable versions.

### 2.4. Data Types

For each type of data, researchers can tailor a HTTP API and trade-off access speed, storage space, versioning support, and ease of programming.

DVID provides a well-defined interface to data type code that can be easily added by users. A DVID server provides HTTP and RPC APIs, versioning, provenance, and storage engines. It delegates data type-specific commands and processing to data type code. As long as a DVID type can return data for its implemented commands, we don't care how it is implemented.

By modifying or adding DVID data type implementations and writing layers over existing storage systems, DVID allows customizable actions on data via a HTTP API. We can tune key-value representations for acceptable performance among storage space, access speed, and ease of programming trade-offs. Different types of checksum and compression can be used for each data type at the key-value level. And we can choose among the different key-value stores and assign the best match for each data instance. For example, for highly compressed label data, we can choose fast but relatively small NVMe SSDs to maximize access speeds.

DVID supports a variety of data types including the following:

**uint8blk**: 3d grayscale volumes.

**labelmap**: 64-bit label 3d volumes, including multi-scale support and sparse volume operations.

**imagetile**: multiscale 2d images in XY, XZ, and YZ orientation, similar to quadtrees.

**annotation**: 3d points that can be accessed by associated label, tags, or spatial coordinate.

**roi**: regions of interest represented via a coarse subdivision of space using block indices.

**keyvalue**: a simple key-value pair store that can be used as a versioned file system.

Each of the data types above use key-value pairs in different ways. While **uint8blk** and **labelmap** both partition 3D space into smaller blocks, the **labelmap** data type persists highly compressed 64-bit supervoxel identifiers in the blocks and also maintains other key-value pairs for label (i.e., neuron identifier) indexing that describe the blocks and supervoxels within any given label. The **annotation** data type can employ up to three different classes of key-value pairs holding JSON-encoded points (synapses, bookmarks, etc.) sorted by ZYX block coordinate, underlying label, and arbitrary string tag. The **keyvalue** data type is a simple pass-through to the underlying key-value store. It is typically used for new types of data until researchers understand the kinds of requests that will be required and whether a new data type should be built to optimize the handling of those requests.

DVID provides a publish/subscribe mechanism for syncing changes in one data instance with associated data instances. For example, we can declare a *segmentation* instance of data type **labelmap** should be synced with a *synapses* instance of data type **annotation**. If a label in *segmentation* is split or merged with other labels, the mutation will be passed to *synapses*, which then updates its internal indexing used for quickly returning all synapses in a given label.

Users can access a detailed description of each data type's Science API by pointing a web browser to a running DVID's HTTP interface. For example, the interface to the **uint8blk** data type can be examined by browsing http://emdata.janelia.org/api/help/uint8blk for a DVID server running on port 80 of **emdata.janelia.org**. Any supported data type can be reviewed by replacing the last word in the help URL with the data type name. Since a detailed exploration of each data type is beyond the scope of this paper, we provide a sampling of the Science API in Table 1 and refer readers to the embedded data type documentation in the DVID github repository.

### 2.5. Versioning 3D Label Data

Each DVID data type provides its own portion of Science API and method of translating the necessary data into key-value pairs. In this section, we describe how data types can evolve by describing the history of four 3D label data types: **labelblk**/**labelvol**, **labelarray**, and finally **labelmap**. The implementation of each data type impacts the speed of neuron editing, the storage efficiency of versioning, and the functions available through its Science API.

The first 3D label data types were **labelblk** and **labelvol**, which handle 64-bit label arrays and each label's sparse volume representation, respectively. The **labelblk** data type allows many ways to read and write the 64-bit unsigned integer label at each voxel. These include reading 2D slices in XY, XZ, and YZ orientation in a variety of formats (e.g., PNG or JPG), reading 3D subvolumes as label arrays in any supported compression scheme (uncompressed, lz4, gzip and/or Neuroglancer's compression format), querying single or multiple voxel coordinates using JSON, and even returning 2D PNG color images where each label has been hashed to a color. For maximum throughput, we also allow reading by blocks so that little processing is necessary and data is streamed from the underlying key-value store to the HTTP connection. The **labelvol** data type allows reading and editing sparse volumes for labels. Its Science API allows reading arbitrarily clipped sparse volumes using run-length encoding (RLE) with optional lz4 or gzip compression. Sparse volumes can also be split or merged.

These first data types only support two scales: the original ingested voxels or “coarse” volumes where each block of voxels was downsampled to a single voxel. Internally, the **labelblk** data type persists key-value pairs where each type-specific key is a ZYX block coordinate that corresponds to the label array for that block. The **labelvol** uses a type-specific key composed of the 64-bit unsigned integer label prefixed to the ZYX block coordinate, and the associated value is the sparse volume RLEs within that block. By doing a range query on a label, the data type code can easily retrieve all RLEs for a given label as well as clip sparse volumes by Z coordinate.

As described in the section above, instances of these two data types can be synchronized using DVID's internal publish/subscribe mechanism. Let us assume that a DVID server is operating on port 8000 of the server *mydvid.net* with a single version at UUID ee78982c87b14d008bb3f93e9e546c10. A two-way sync can be established between a *segmentation* instance of **labelblk** and a *sparsevol* instance of **labelvol** by sending a JSON string *{“sync”:“segmentation”}* to *http://mydvid.net/api/node/ee789/sparsevol/sync* and a reciprocal string to *http://mydvid.net/api/node/ee789/segmentation/sync*. Note that HTTP requests only need a recognizable substring of a UUID rather than the full 32 character hexadecimal string.

If a user merges two labels via the **labelvol** merge request, a synced **labelblk** instance will automatically modify all voxels affected by the merge. Similarly, if the labels of voxels are modified through **labelblk** instance requests, the change will be sent to the synced **labelvol** instance and the sparse volumes of any affected label will be modified.

We could also sync a *synapses* instance of **annotation** data type with the underlying label volume by sending a JSON string *{“sync”: “segmentation”}* to *http://mydvid.net/api/node/ee789/synapses/sync*. This one-way sync means changes in the label volume will automatically modify the list of synapses corresponding to the affected labels.

While the first iteration of 3D label data types was successful and allowed very fast retrieval of sparse volumes due to its separate storage, we found that maintaining the sparse volumes using our admitedly simple format could dominate the underlying key-value store. So we created the **labelarray** data type that consolidated both **labelblk** and **labelvol** Science APIs under one implementation without the need for syncs.

The **labelarray** data type supports multi-scale representation and primarily uses two classes of key-value pairs as described earlier in section 2.3: label data organized into blocks and label indices describing the blocks intersected by each label. The storage requirement is significantly smaller than synced **labelblk**/**labelvol** instances because potentially large sparse volumes are replaced by an index of blocks. The new data type also exhibits faster write and much slower sparse volume read times since precomputed RLEs are not stored but must be computed on-the-fly.

The most recent **labelmap** data type adds in-memory label maps to the **labelarray** architecture and uses supervoxel identifiers as the block label data. Many segmentation techniques generate an initial base segmentation that tends to be conservative followed by more aggressive agglomeration passes (Nunez-Iglesias et al., 2014; Parag et al., 2015; Januszewski et al., 2018). The **labelmap** data type supports this approach. By using an in-memory label map, label merges are extremely fast and do not alter the underlying supervoxel blocks. We also allow “cleave” operations that split label bodies along supervoxel boundaries, thereby preserving underlying supervoxel blocks as well and only modifying the label map. Supervoxel splits require modification of the block key-value pairs but are relatively rare compared to merges and cleaves, particulary as both the underlying grayscale imaging and automatic segmentation processes improve.

For each node in the DAG, the **labelmap** data type stores label edits (merges, cleaves, and supervoxel splits) in an append-only log. Requests can cause lazy loading of all edits from the root to the given version and population of the in-memory label map.

The newer **labelmap** and **labelarray** data types store label data in a highly compressed format inspired by the Neuroglancer compression scheme^12^, which partitions each block of data into smaller sub-blocks. The DVID label compression format makes the following changes: (1) adds a block-level label list with sub-block indices into the list (minimal required bits vs 64 bits per index in the original Neuroglancer scheme), and (2) the number of bits for encoding values is not required to be a power of two. A block-level label list allows easy sharing of labels between sub-blocks, and sub-block storage can be more efficient due to the fewer bits per index (at the cost of an indirection) and better encoded value packing (at the cost of byte alignment). We gain space, up to an additional 2x compression for a given block, and simpler label updating at the cost of increased computation and Neuroglancer's explicit GPU support. Although label blocks are stored in this highly compressed format, data can be transcoded to Neuroglancer's compressed segmentation format during requests.

Despite how differently the four data types implement 3D label support, the HTTP APIs are mostly identical save for optional features that were added in later data types.

### 2.6. Storage Backends

The use of key-value storage (KVS) systems as the underlying store brings a number of benefits. Open source KVS systems span from simple, embedded leveldb implementations to strongly-consistent, globe-scale distributed systems. Once data is immutable, there are number of distributed KVS systems for efficiently caching the data (e.g., groupcache). This allows us to build branched versioning systems that use different kinds of KVS systems for different classes of data. For example, relatively immutable, very large data like original grayscale imaging can be assigned to extremely scalable cloud-based systems and cached locally using off-the-shelf software due to its immutable nature, while much more compressible and mutable data like segmentation can be stored on fast NVMe SSD drives.

**Figure 7:**
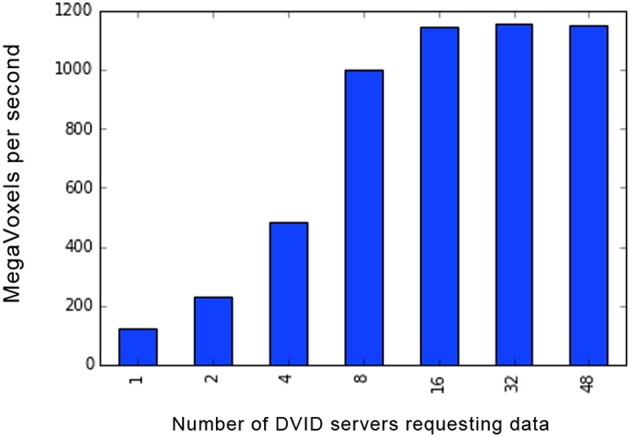
Scalability of uncompressed grayscale image reads from Google Cloud Store backend. As the number of DVID servers increase, simultaneously requesting non-overlapping image subvolumes from a 16 TeraVoxel dataset, the throughput plateaus just below 1.2 Gigavoxels or 9.6 Gigabits per second. Servers were at the Janelia cluster with 16 real request threads per server, connecting to a Northern Virginia Google Cloud Store through a 10 Gigabits per second connection. The grayscale instance had only one version corresponding to the ingested image (8-bit/voxel) volume.

Figure 7 shows the scalability of the Google Cloud Store as a backend for immutable, uncompressed grayscale (unsigned 8-bit intensity per voxel) volumes. Because the data is immutable, any number of DVID servers can be spun up and directed toward the cloud. The maximum throughput using test servers at Janelia requesting data from Google Cloud Storage is slightly less than 1.2 Gigavoxels (9.6 Gigabits) per second, which corresponds to the 10 Gigabit per second connection from Janelia to the internet. If we were looking through a sequence of grayscale images, this would amount to approximately 4,400 (512 × 512 pixel) images per second, or 73 proofreaders scrolling through those images at 60 fps.

Currently, the bulk of the FlyEM Team's reconstruction and segmentation data is held in leveldb databases on NVMe solid state drives and cheaper RAID-10 systems with hard disk drives. Newer grayscale volumes are kept in Google Cloud Store and we are experimenting with a simple key-value interface to the file system. Contributors have recently added a storage engine for OpenStack Swift.

### 2.7. Availability

DVID is freely available on github (http://github.com/janelia-flyem/dvid) under the Janelia Open-Source Software license. The wiki section of that github repository provides user guides as well as installation instructions for pre-built binaries, conda builds, and docker containers.

## 3. Related Work

Typically, researchers have dealt with image-oriented data by either storing it in files or writing software systems that use a relational database to store image chunks or file pointers. Connectomics data servers include bossDB (Kleissas et al., 2017), OpenConnectome (Burns et al., 2013), CATMAID (Saalfeld et al., 2009), and more visualization-focused systems like BUTTERFLY (Haehn et al., 2017). DVID is distinguished from these other systems by its support of branched versioning, an extensible Science API through data type packages, and extremely flexible storage support through a variety of key-value store drivers.

The first to support branched versioning at large scale was SciDB (Stonebraker et al., 2011). An approach to branched versioning in relational databases culminated in OrpheusDB (Huang et al., 2017). Both SciDB and OrpheusDB could be used as storage backends for DVID data types that match their strengths. For example, SciDB is particularly adept at handling multi-dimensional arrays and could be used for the voxel data component of DVID label data types, while OrpheusDB could be used for heavily indexed synapse point annotations.

The DataHub effort (Bhardwaj et al., 2014) has very similar aims to bring a distributed versioning approach to scientific datasets, offering an analog to github.com with a centralized server that builds on a Dataset Version Control System (DVCS). DataHub and DVID developed in parallel and focused on different types of data. DVCS was designed to handle datasets in the sub-Terabyte range without an emphasis on 3d image data, and it's API is a versioning query language based on SQL so the significant connectomics-focused data layer would still be needed. Much as OrpheusDB is a possible storage engine for smaller data types like annotations, DVCS could be considered a possible storage interface to DVID.

Ideally, connectomics tools would be able to use a variety of data services. This would require the community to develop common interfaces to standard operations. Currently, simple operations like retrieving 2D or 3D imagery are sufficiently similar across services so that tools like CATMAID, Neuroglancer, and BigDataViewer (Pietzsch et al., 2015) can use different image volume services including DVID.

## 4. Future Work

Distribution of versioned data can help to efficiently synchronize remote servers, a significant problem given the scale of VEM data (Lichtman et al., 2014). For example, when establishing remote copies of massive image volumes, we envision shipping one or more disks and then synchronizing servers by sending only data associated with new nodes in the version DAG. The speed of such operations depend on the ability to easily extract and transmit data from a subset of versions as well as fast mechanisms for moving data between servers. Our current version-last approach to key encoding makes version-based transmission costly, since it requires scanning all keys. As the FlyEM Team increases our sharing of reconstructions to researchers around the world, we expect to spend some energy to improve data transfer rates and how version data is organized.

**Figure 8:**
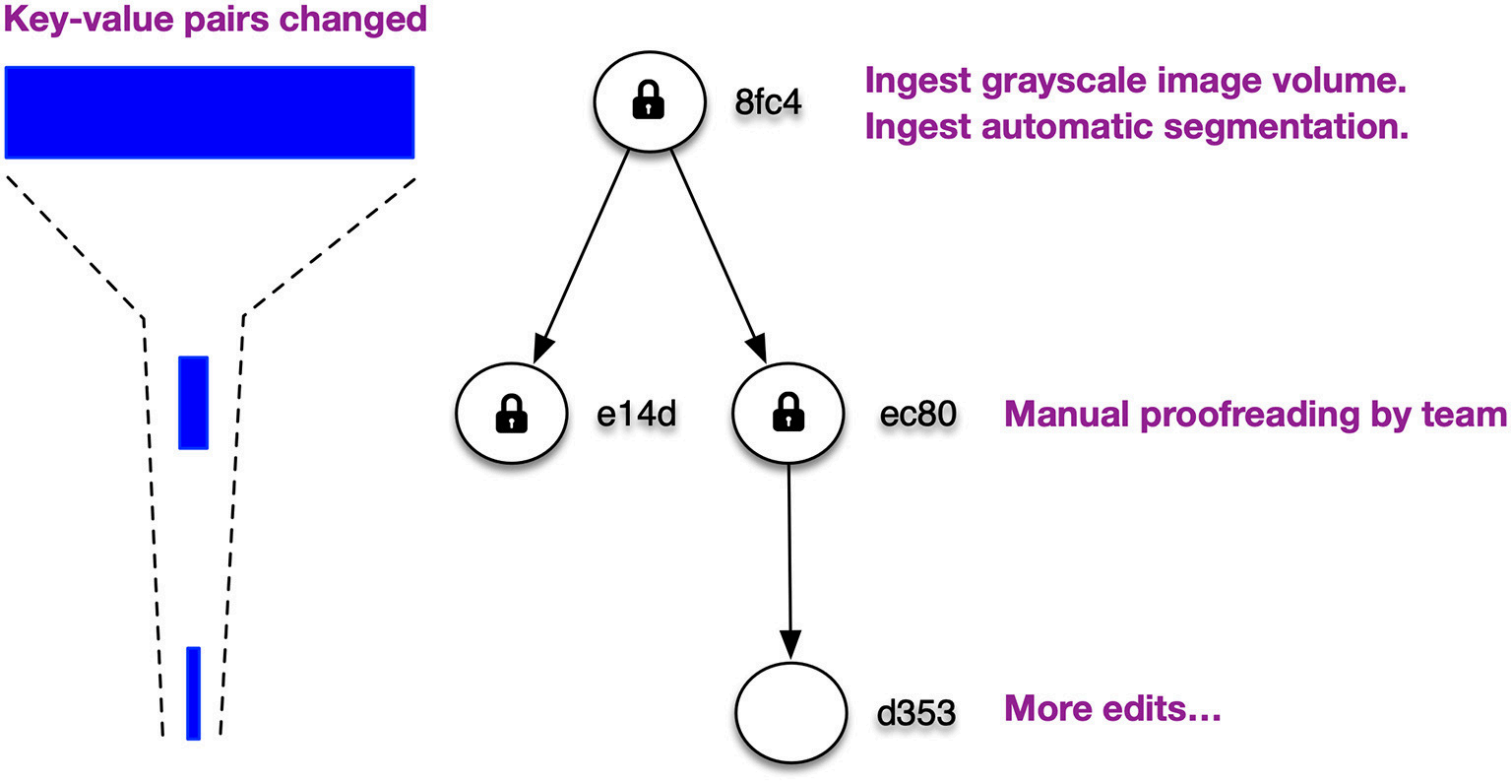
Typical EM reconstructions produce a version DAG with most changes toward the root and fewer, human-guided changes toward the leaf nodes. This means that the bulk of data will be committed and immutable.

One such effort is a petabyte-scale DVID-tuned datastore now in the planning stages. Mutations are relatively expensive since they generally require transactions and impose difficult coordination issues when scaling operations to multiple servers. Immutable data storage is simpler and can be accelerated through a variety of techniques. For VEM reconstructions at Janelia, the majority of data exists near the top of the DAG since most of our workflows involve ingesting very large image volumes and pre-generating segmentation for every voxel (Figure 8). This suggests a multi-stage store where on initial ingestion and subsequent version commits, the committed, immutable data is transformed to optimize reads, storage size, and ease of version-based distribution.

The bulk of our data can persist in immutable stores that combine compact, in-memory key indexing with version-first, append-only file storage, suitable for easy access and transmission of version deltas. This allows us to use smaller, faster storage solutions for the mutable portion of the DAG, namely the leaf nodes where manual editing tends to dominate. Retrieval of data from any version then requires concurrent retrieval from both the immutable and mutable stores.

**Figure 9:**
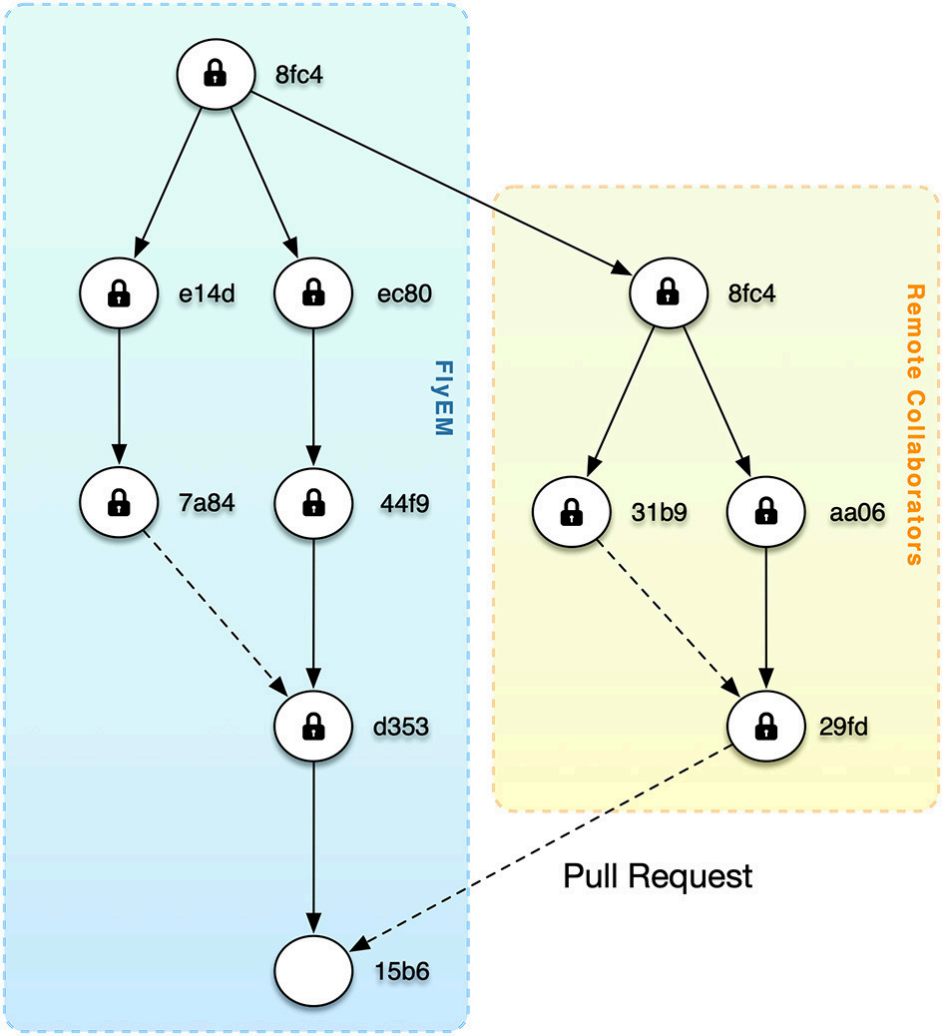
As shown by software version control systems like git, distributed versioning is an effective workflow for sharing changes via pull requests. The figure depicts a future scenario where the root version at Janelia has been shared with remote collaborators. After changes at the remote site, a pull request is sent back.

We want to enable researchers to work on their own branches, optionally limit download to regions of interest, and share changes via pull requests (Figure 9). This is particularly appealing when considering the publication of massive datasets where specialists may improve regions and submit changes.

Currently, DVID provides branched versioning that meets the needs of most of our current reconstruction workflow. Only some work has been done on the remote distribution and syncing aspects corresponding to the *push* and *pull* operations of distributed versioning systems like *git*. DVID can push data to remote repositories and merge nodes using simple conflict resolution like node A always wins against node B if there is a conflict. In order to allow more sophisticated merges, we need to add data type-specific merge tools to the DVID ecosystem. For example, when merging two nodes of segmentation, we would want a merge tool to provide visualizations of conflicts and allow a user to choose a proper merge result. DVID should be agnostic to the form of the merge tool yet provide a conflict resolution API that could be used to select conflicts and post results.

Availability of a merge tool also allows the possibility of scaling proofreading by using entirely separate DVID servers instead of scaling up a single DVID server.

Versioning should allow downloading portions of massive datasets since it can reference the originating UUID. While full datasets may require large servers with many terabytes of high-speed storage, we plan to facilitate proofreading of regions of interest on laptops even in an offline setting. This would be similar to standard git workflows where programmers modify code locally and then submit pull requests of their changes to the central server.

In the near future, we plan on adding Badger^13^ and RocksDB as drop-in replacements for the current leveldb storage backend.

Although DVID has initially focused on key-value stores, we are evaluating OrpheusDB (Huang et al., 2017) and may eventually support fundamentally different types of stores (*polyglot persistence*) like graph, relational, and scientific array databases. We are currently investigating OrpheusDB as a backend for the DVID synapse annotation data type, which indexes synapse point annotations across space, assigned labels, and arbitrary tags. Unfortunately, *polyglot persistence* comes at the cost of increased code to extend operations like remote distribution beyond simple key-value pairs to these new types of stores.

## 5. Conclusions

The DVID system is a powerful tool that allows us to immediately view our dataset at any commit time, and also enables training of proofreaders so that they can handle large connectomes. It has allowed us to flexibly store very large immutable datasets in the cloud in conjunction with fast, smaller storage for mutable data. This has allowed us to scale our operation and provide regional data services to collaborators. More importantly, we feel that distributed versioning in connectomics could be an extremely powerful tool for collaborating with researchers around the world. As the amount of published data increases dramatically due to advances in imaging, segmentation, and reconstruction workflows, there will be an increasing need to provide provenance and mechanisms for collaborative data editing and analysis. Just as distributed versioning with its notion of pull requests has greatly impacted the open source software movement, we believe that it can alter the way we think of sharing and editing connectomics data.

## Author Contributions

WK designed and implemented the core DVID system. SP helped design DVID and implemented a labelgraph data type and the Google Cloud Store engine.

### Conflict of Interest Statement

The authors declare that the research was conducted in the absence of any commercial or financial relationships that could be construed as a potential conflict of interest.

## Footnotes

^1^http://github.com/janelia-flyem/dvid

^2^https://martinfowler.com/articles/richardsonMaturityModel.html

^3^https://httpie.org/

^4^https://github.com/janelia-flyem/dvid-console

^5^https://github.com/janelia-flyem/libdvid-cpp

^6^https://github.com/janelia-flyem/DVIDSparkServices

^7^https://github.com/google/neuroglancer

^8^https://tools.ietf.org/html/rfc4122

^9^http://leveldb.org

^10^https://rocksdb.org

^11^https://cloud.google.com/storage/

^12^https://goo.gl/LNMLJo

^13^https://github.com/dgraph-io/badger
